# The biology of mental pain: a systematic review to map the different expressions, definitions, hypotheses, experimental paradigms, investigation methods and candidate biomarkers of mental pain in human subjects

**DOI:** 10.1192/j.eurpsy.2024.571

**Published:** 2024-08-27

**Authors:** E. K. Duranté, A. Ribeiro, L. Gaspard-Boulinc, C. Lemogne, I. Boutron, A. Chevance

**Affiliations:** ^1^METHODS Team, Université de Paris Cité, CRESS, INSERM UMR 1153; ^2^Centre d′Épidémiologie Clinique, Hôpital Hôtel Dieu, AP-HP; ^3^IBENS, École Normale Supérieure; ^4^Department of Psychiatry, Hôpital Hôtel Dieu, AP-HP, Paris, France

## Abstract

**Introduction:**

Mental pain is a transdiagnostic symptom, predictive of suicide and reported as a critical outcome by patients. A previous systematic review of epidemiological and clinical research has shown a lack of consensual definition of mental pain in clinical research and high heterogeneity across the different measurement instruments of mental pain. Up today there is no systematic review synthetizing all published biological investigations on mental pain.

**Objectives:**

This study aims to map the field of biological investigations of mental pain in human to identify what and how biomarkers are investigated with a meta-research approach, by providing a critical appraisal of the terms and definitions of mental pain, the studies’ hypotheses, the experimental paradigms used to induce or mimic mental pain and the measurement instruments used to measure mental pain.

**Methods:**

We conducted a systematic review (compliant with PRISMA guidelines) of all primary research reporting to investigate candidate biomarkers of mental pain in human subjects as stated by the authors. We searched from inception to June 23^rd^, 2022, the 3 databases MEDLINE, Web of Science and EMBASE. We extracted the study characteristics (e.g., year of publication, population, etc.), the terms used for meaning mental pain, the definition of mental pain, the method to induce mental pain and its rational, the hypotheses and aims, the measurement instruments of mental pain, the candidate biomarkers, and their method of investigation. We performed descriptive statistics of the sample’s characteristics and the extracted data, a qualitative analysis of the definitions, hypothesis, aims and experimental paradigms, and a data visualization linking candidate biomarkers, experimental paradigms, and their investigation methods.

**Results:**

The search retrieved 5685 papers of which we included 72 primary research publications constituting 78 distinct research studies. Only 37.5% of studies reported a definition of mental pain. 11.5% of studies did not show a measurement instrument of mental pain. The Cyberball (a social exclusion paradigm) was the most frequently used paradigm in experimental studies (62.7%). The cingulate cortex was the most frequently investigated biomarker category (15.3% of all candidate biomarkers), with fMRI as the most frequent investigation method (53.7% of all investigation methods).

**Conclusions:**

The field of biological investigations on mental pain shows a marked heterogeneity of definitions, terms, hypotheses, experimental paradigms, and measurement instruments, with an over-representation of the construct of social pain and the Cyberball. These could compromise the comparison and combination of studies results in evidence synthesis and their translation into clinical practice.

**Disclosure of Interest:**

None Declared

